# Transylvania School of Dental Surgery

**Published:** 1850-10

**Authors:** 


*Transylvania School of Dental Surgery.*—From a notice and advertisement in the "Progress of the Age," a newspaper published in Lexington, Ky., we learn that a school of dental surgery, under the above title, chartered by the legislature of Kentucky, 1850, will commence its first session on the first Monday in December.

The faculty is composed of:

J. B. Stout, M. D., of Lexington, Ky., Professor of the Institutes of Dental Science; John B. Lindsay, D. D. S., of Flemingsburg, Ky., Professor of the Principles and Practice of Dental Surgery; B. J. Dudley, D. D. S., of Maysville, Ky., Professor of Operative and Mechanical Dentistry; Jas. S. Drane, M. D., of New Castle, Ky., Professor of Special Anatomy, Physiology and Pathology; and Dr. G. W. Evans, of Lexington, Ky., Professor of Dental Chemistry, Hygiene and Therapeutics.

Candidates for graduation are required to attend two courses of dental lectures, the last to be in this institution, or one course of medical lectures and one course in this school; and practitioners of dental surgery, of good standing, upon satisfactory examination, are entitled to the same privilege.

Although we have but little knowledge of the competency of the faculty to teach, the editor of the paper containing the advertisement, speaks of the prospects of the school in the most confident terms, and of the scientific attainments and practical skill of the gentlemen who occupy the several chairs in the institution.

